# Ulnar Osteotomy with 2-Pin Unilateral Gradual Distraction for Treatment of Chronic Monteggia Fracture: A Case Report

**DOI:** 10.5704/MOJ.1703.015

**Published:** 2017-03

**Authors:** SG Gooi, CS Wang, A Saw, O Zulkiflee

**Affiliations:** Department of Orthopaedics, Pulau Pinang Hospital, Georgetown, Malaysia; * National Orthopaedic Center of Excellence for Research and Learning (NOCERAL), University of Malaya, Kuala Lumpur, Malaysia

**Keywords:** chronic monteggia, monotube, gradual lengthening

## Abstract

Missed Monteggia fracture leading to chronic radial head dislocation is a known complication. The surgical treatment options remain challenging. The aim of treatment is to reduce the radial head and to maintain the stability of the elbow in all ranges of motion. A few surgical techniques have been described with complications. We report the case of a 13 years old boy with chronic radial head dislocation as a result of an unrecognised Monteggia fracture-dislocation for eight years. We successfully reduced the radial head and corrected the cubital valgus from 45 degrees to 10 degrees with a proximal ulna osteotomy and gradual distraction with 2-pin Monotube external fixator. The correction was uneventful with good functional outcome.

## Introduction

A missed diagnosis of Monteggia fracture-dislocation is a known complication. The treatment of chronic radial head dislocation following missed Monteggia remains controversial. Generally, the duration of missed diagnosis determines the technical difficulty of the surgery and postoperative clinical function. The key problem of missed Monteggia is the malunited ulnar bone leading to the irreducible radial head. The aim of surgery is reduction of the radial head and maintaining the stability in all ranges of motions. Several surgical techniques have been proposed, namely annular ligament reconstruction, corrective osteotomy of the ulna with internal fixation and bone graft, and corrective osteotomy of the ulna with external fixator ^1^-^3^. Reported complications for this challenging procedure include subluxation and re-dislocation, elbow instability and stiffness, non-union of osteotomy, avascular necrosis of the radial head, nerve injury and infection^4^.

## Case Report

**Fig. 1:**
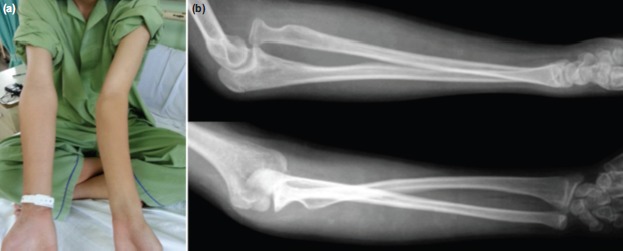
(a) Pre-operative clinical picture with cubital valgus of 45 degree and (b) Pre-operative radiograph with radial head dislocated anteriorly.

We report a 13 years old boy, who had a fall at age of five years. Following the fall, he complained of left forearm pain and was brought to hospital. He was treated conservatively with Plaster of Paris. Post-injury over the years the left upper limb deformity was noted to progress gradually. In view of tolerable pain initially and only mild angulation of the elbow, the parents did not seek medical attention for the child. However, over the years, the elbow pain progressively worsened with increasing deformity, affecting his daily activities. He presented to our clinic at age of 13. On examination, there was left cubital valgus deformity of 45 degrees without ulnar nerve neuropathy (Fig. 1a). The elbow range of movement was full. On palpation, radial head was felt at the anterior elbow. Radiographic assessment revealed anterior dislocation of radial head, Bado type 1^5^ (Fig. 1b).

Surgical option was offered to the patient. We performed left ulna osteotomy and gradual lengthening using 2-pin uniplanar external fixator (Monotube). Only one Schanz pin proximally and one distally were inserted from the osteotomy site. No transfixing shanz-pin or k- wire across radius/ulnar distally. Post-operative recovery was uneventful. Gradual distraction was started at day 7 postoperative. Patient was followed-up at two weekly intervals with radiography of the elbow and forearm. At seventh postoperative week, the, radial head was reduced with approximately 3 cm ulna lengthening. Hence the distraction process was stopped.

**Fig. 2:**
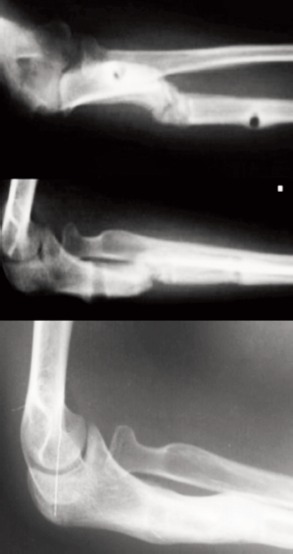
Radiograph at 3 months follow-up. Reduced radial head and osteotomy site united. At one year follow-up, radial head remain reduced.

At three months post-surgery, the lengthening bone gap consolidated. The external fixator was removed and an above elbow backslab was applied for another month. After removal of the cast, all wounds had healed. Clinically, cubital valgus had reduced to 10 degrees, the ranges of motion were full for both supination/pronation and flexion/extension. Radiographs of left elbow showed reduced radial head and corticotomy site consolidated (Fig. 2). At one year follow-up, radial head remained well reduced and stable in all ranges of elbow motion.

## Discussion

In chronic Monteggia fracture-dislocation, the mal-united ulnar leads to radial head dislocation. Dislocated radial head causes lateral elbow joint restraint deficiency, leading to gradual cubital valgus. Our aim was to reduce the dislocated radial head and at the same time maintain its stability while allowing correction of the ulna.

In the surgery we performed there was only one Schanz pin inserted proximally to the osteotomy site and the other one distal to it. This less rigid construct allowed natural ulna angulation during the process of lengthening while expecting the radial head to reduce spontaneously. The lack of stability of this construct was not worrisome as the forearm has paired bones held by strong interosseous membrane. Furthermore, forearm is not a weight bearing limb. Hence, at the end of distraction osteogenesis, when the radial head had reduced into the desired place, the lengthened ulnar bone was angulated and not in a same plane. This resembled the bending of the ulnar bone in cases where the surgeon performs concurrent correction with osteotomy and plating of the ulna^1^, ^2^. Exner^3^ reported gradual ulna distraction with external fixator for radial head reduction without opening the joint. He also highlighted the lengthening of the ulna with angulation as the key to maintaining the reduction of the radial head.

In our case, there was also no transfixing shanz-pin or k-wire across radial bone and ulnar bone distal to the level of corticotomy. We believe that the strong interosseous membrane between the ulnar and radial is able to bring down the radial head as the ulnar bone is being lengthened. With this concept, with no fixation between the two bones, the patient was allowed to supinate and pronate the forearm during the whole process. This prevented the complication of limited pronation of the forearm which is very common.

We did not open up the radio-capitellar joint for open reduction or annular ligament reconstruction, as in our case, eight years post trauma may be too complicated as all structures around that region would have healed with fibrosis. Alexendre *et al*^2^ found that reconstruction of the annular ligament was unnecessary, as most the radial heads were stable without reconstruction. Moreover, the dissection to reconstruct the annular ligament will subject the patient to complications such as elbow stiffness, avascular necrosis of the radial head, heterotopic ossification and radio-ulnar synostosis^1^,^4^.

We concluded that missed or neglected Monteggia fracture-dislocation with chronic radial head dislocation can be treated with simple proximal ulna osteotomy and gradual distraction osteogenesis of ulna via a minimally invasive surgery using uniplanar mono-tube external fixator. The functional and radiological outcomes are good in the short term follow up of this patient.
